# Effects of 14-day oral low dose selenium nanoparticles and selenite in rat—as determined by metabolite pattern determination

**DOI:** 10.7717/peerj.2601

**Published:** 2016-10-20

**Authors:** Niels Hadrup, Katrin Loeschner, Kasper Skov, Gitte Ravn-Haren, Erik H. Larsen, Alicja Mortensen, Henrik R. Lam, Henrik L. Frandsen

**Affiliations:** 1 Division of Toxicology and Risk Assessment, National Food Institute, Technical University of Denmark, Søborg, Denmark; 2 Division for Food Technology, National Food Institute, Technical University of Denmark, Søborg, Denmark; 3 Division of Food Chemistry, National Food Institute, Technical University of Denmark, Søborg, Denmark; 4 Division for Diet, Disease Prevention and Toxicology, National Food Institute, Technical University of Denmark, Søborg, Denmark; 5 National Research Centre for the Working Environment, Copenhagen, Denmark; 6 Department for Environment and Toxicology, DHI, Hørshom, Denmark

**Keywords:** Metabolomics, Toxicology, Pharmacology, Selenium, Nanoparticle, Metabolomic pattern recognition

## Abstract

Selenium (Se) is an essential element with a small difference between physiological and toxic doses. To provide more effective and safe Se dosing regimens, as compared to dosing with ionic selenium, nanoparticle formulations have been developed. However, due to the nano-formulation, unexpected toxic effects may occur. We used metabolite pattern determination in urine to investigate biological and/or toxic effects in rats administered nanoparticles and for comparison included ionic selenium at an equimolar dose in the form of sodium selenite. Low doses of 10 and 100 fold the recommended human high level were employed to study the effects at borderline toxicity. Evaluations of all significantly changed putative metabolites, showed that Se nanoparticles and sodium selenite induced similar dose dependent changes of the metabolite pattern. Putative identified metabolites included increased decenedioic acid and hydroxydecanedioic acid for both Se formulations whereas dipeptides were only increased for selenite. These effects could reflect altered fatty acid and protein metabolism, respectively.

## Introduction

Selenium (Se) is an essential element for humans. Se is a building block of the amino acid selenocysteine, which is necessary in the synthesis and catalytic function of selenoproteins such as peroxidases and reductases (Levander, 1982; Bulteau & Chavatte, 2015). At high doses, Se becomes toxic. Neurological effects in humans, were reported after the ingestion of a nutritional supplement at a dose corresponding to 60 to 120 μg/kg bw/day for 14 days (chemical form not reported) (Clark et al., 1996). Human Se poisoning involving mental disturbances was reported with an estimated intake of 0.34 mg/kg bw/day for 6 weeks (chemical form not specified) (Sutter et al., 2008). Human mortality was observed after the ingestion of 10 g of sodium selenite (See et al., 2006; Williams & Ansford, 2007). In addition, the ingestion of selenous acid (in the form of gun-bluing agent) has been associated with several fatal human intoxications (Pentel, Fletcher & Jentzen, 1985; Matoba et al., 1986; Hunsaker, Spiller & Williams, 2005).

To provide more effective Se dosing regimens, nanoparticle formulations have been developed. The concept is that the Se nanoparticles provide a slow release of Se ions, thereby reducing acute toxicity. A few studies have demonstrated lower toxic potency of Se nanoparticles than of dissolved ionic Se species. This suggests that to some extent, Se from nanoparticles is less bioavailable (Zhang et al., 2001; Zhang et al., 2005; Jia, Li & Chen, 2005; Benko et al., 2012). However, Se nanoparticles, with oxidation state 0, may also exert biological and toxicological effects different from ionic Se formulations with other oxidation states. In addition, nanoparticles have a very large surface to volume ratio and are known to bind to e.g., proteins, and reactions may be catalyzed by the nanoparticle surface (Klein, 2007). Therefore, nanoparticles may have different toxic properties as compared to ionic species. Thus, to avoid deleterious effects in humans it is important to determine if selenium nanoparticles exert biological and/or toxic effects different from those exerted by selenium ions. The Se nanoparticles are intended for humans in doses at which overt toxicity e.g., body weight loss is not expected to occur. Thus, sensitive techniques measuring a large number of parameters are needed to provide a broad screen of potential effects. Metabolite pattern determination draws on the concept of metabolite profiling (metabolomics) to enable the concomitant measurements of a large number of metabolites (Robertson, Watkins & Reily, 2011). Using metabolite pattern determination on a body compartment such as urine or blood plasma, differences in the biological effect profiles of chemical substances can be assessed. This is done by comparing the identities and levels of metabolites altered by each substance, and potentially linking them to biological and toxicological mechanisms of action.

In the present study, we investigated whether Se nanoparticles at low dose exert biological or toxicological effects, which are different from the effects caused by dosing with ionic selenium. For this, we used metabolite pattern determination on urine from rats dosed equal amounts of Se formulated as nanoparticles (oxidation state 0) or for comparison as selenite ions (oxidation state IV). We used LC-MS to analyze urine samples obtained after 14 days of oral administration of Se nanoparticles (19 nm in mean diameter) or sodium selenite in doses of 0.05 and 0.5 mg Se/kg bw/day. The maximum safe dose in humans is 300 μg/day (5 μg/kg bw/day) (Scientific Committee on Food, 2006). Thus, these doses correspond to 10 and 100 fold, respectively of that dose; or 1.5 and 15 fold that dose when adjusting for body surface area (Reagan-Shaw, Nihal & Ahmad, 2008). These doses were selected in order to investigate the effects of Se in the range of human essential doses to borderline toxicity doses. It is in this range that Se from nanoparticles will act in case of excessive Se release in humans. Regarding the relevance of these doses to rat toxicity levels, the highest dose selected was at or just below doses giving slight reduction in body weight in previous rat studies (Palmer & Olson, 1974; Cabe, Carmichael & Tilson, 1979; Dausch & Fullerton, 1993; Raines & Sunde, 2011). Analyses of the urinary metabolite patterns of rats dosed Se nanoparticles or selenite were comparable. Identified likely metabolite candidates included increased decenedioic acid and hydroxydecanedioic acid for both Se formulations, whereas dipeptides were only increased for selenite. These effects could reflect altered fatty acid and protein metabolism, respectively.

## Materials and Methods

### Nanoparticles

**Figure 1:**
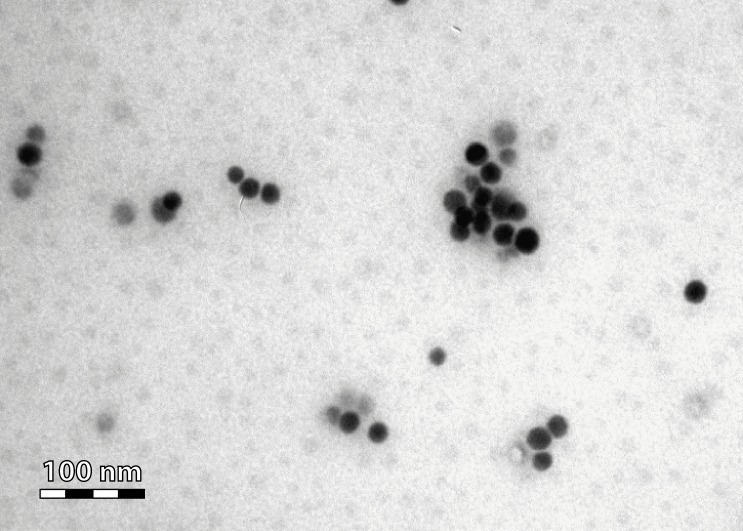
TEM micrograph of the administered Se nanoparticles.

Se nanoparticles with a mean diameter of 19 nm (ranging in size from 10 to 80 nm) were produced, stored and characterized as previously described (Zhang et al., 2001; Loeschner et al., 2014). Briefly, the 19 nm nanoparticles were synthesized by reduction of sodium selenite with glutathione in the presence of bovine serum albumin (BSA). BSA was added as a stabilizing agent as nanoparticles tend to aggregate and eventually precipitate. The concentration of Se in the nanoparticle suspension was determined following digestion by nitric acid by inductively coupled plasma mass spectrometry (ICP-MS). The nanoparticle size distribution was determined by dynamic light scattering (10–80 nm). The Se in the nanoparticles had an oxidation state of 0. Images to assess the size and shape of the Se nanoparticles were generated by transmission electron microscopy (TEM) (Fig. 1) using a TEM Philips CM100 instrument (FEI, Eindhoven, The Netherlands) at 80 kV accelerating voltage (Loeschner et al., 2014).

### Animal study

Urine samples were obtained from a previously reported Se bio-distribution study (Loeschner et al., 2014). Briefly, four-week-old, specific pathogen-free (SPF) female Wistar rats were obtained from Taconic M&B (Lille Skensved, Denmark). The rats were allowed to acclimatize for one week. The rats were housed in pairs with a 12:12-h light/dark cycle with the lights on from 7 a.m. to 7 p.m. The room temperature was 22 ± 1°C, and the relative humidity was 55% ± 5%. The rats were given ad libitum access to a standard diet (Prod. no.1324; Altromin International, Lage, Germany) and citric acid acidified tap water. The animals were randomized by weight. The test substances were administered by oral gavage in a volume of 10 mL/kg bw once a day for 14 days. The treatment groups were as follows: 1. Vehicle-BSA (bovine serum albumin 4 g/L) control (*n* = 8); 2. Se nanoparticles 0.05 mg Se/kg bw/day stabilized with BSA (4 g/L) (*n* = 6); 3. Se nanoparticles 0.5 mg Se/kg bw/day (*n* = 6) stabilized with BSA (4 g/L); 4. Vehicle-H_2_O (*n* = 8); 5. Sodium selenite 0.05 mg Se/kg bw/day (*n* = 8); and 6. Sodium selenite 0.5 mg Se/kg bw/day (*n* = 8). No BSA was added to groups 4, 5 and 6; thus, group 4 acted as the control group for the sodium selenite groups. Sodium selenite (Na_2_SeO_3_) was obtained from Sigma-Aldrich (Copenhagen, Denmark). On experimental day 14, the rats were weighed and placed individually in metabolism cages for 24 h for the collection of urine. During the 24-hour period, the urine samples were frozen by collection on dry-ice. Subsequently, the samples were stored at −80°C. In the metabolism cages, the rats had access to drinking water but not to feed. The animal study was conducted under conditions approved by the Danish Animal Experiments Inspectorate (approval number 2004/561-917) and the in-house Animal Welfare Committee.

### HPLC/MS metabolite pattern determination analysis

Metabolite pattern determination of the urine was performed as previously described (Hadrup et al., 2012). In brief, the urine samples were precipitated with two volumes of methanol and centrifuged (10 min at 10,000 × g). The supernatants were collected and analyzed by HPLC coupled to a qTOF-MS. Sample injection volumes were normalized to the creatinine concentration of the urine to adjust for differences in diuresis. The metabolites were separated on an Ascentis Express C8, 2.7 µm, 100 × 2.1 mm column (Supelco, Bellefonte, PA, USA, product no. 53832-U). The initial flow rate was 0.25 mL/min, increased to 0.4 mL/min at 9 min. The solvents were 10 mM ammonium formate (A, Fluka, Seelze, Germany, product no. 70221), pH 3.5, and acetonitrile (B, Fluka, Seelze, Germany, product no. 14261). Solvent programming was 0% B at 0 min followed by a linear gradient to 100% B in 9 min, holding at 100% B at 10 min. The oven temperature was 40 °C. The metabolites were detected by use of a Bruker microTOFq time-of-flight mass spectrometer equipped with an electrospray ion source (Bruker Daltonics, Bremen, Germany). The samples were analyzed in both positive and negative ionization modes. In time segments between 0.2 and 0.4 min sodium formate clusters were introduced in the ion source and these clusters were used for calibration of the data files. The data obtained are reported as the mass-to-charge (m/z) ratios and HPLC retention times of the metabolites. These are given in the format of xxxx.xxx Da, yyy s (seconds).

The analyses of the chromatograms were conducted using the Profile Analysis 2.1 software package (Bruker Daltonics, Bremen, Germany). Data buckets were constructed using a time window from 60 to 720 s with an m/z ratio range of 100 to 700 using the “find molecular feature” algorithm including time alignment. The noise was reduced using R (R Core Development Team, 2014) by removing peaks that were present in <50% of the samples of each treatment groups and had peak intensities of ≤3,000 counts per s (cps). The raw intensity data were next transferred to the online MetaboAnalyst server (Xia et al., 2009). The data were normalized as the sums, and Pareto-scaling was performed. Partial least squares discriminant (PLS-DA) analysis and ANOVA were applied to the BSA-vehicle *vs.* Se nanoparticle groups and separately to the H_2_O vehicle *vs.* sodium selenite groups. Discriminatory metabolites were selected based on a false discovery rate-adjusted p value of 0.05. The corresponding raw data for these metabolites were then transferred to Graph Pad Prism to establish curves and test for normal distributions. Normality was tested using the Kolmogorov Smirnov test (with Dallal-Wilkinson-Lillefor *p*-value). The data (BSA vehicle *vs.* Se nanoparticle groups and H_2_O vehicle *vs.* selenite groups) were then evaluated again by ANOVA or by Kruskal–Wallis depending on the presence or absence of normal distribution. Dunnett’s and Dunn’s post tests were applied to determine the effects of single treatment groups compared to their respective control groups. Although evaluated separately data for both Se nanoparticles and selenite were presented on the same bar graphs for comparison. All data on the graphs are presented as the mean, and the error bars represent SEM. A false discovery rate-corrected *p*-value of less than 0.05 was considered significant.

The metabolites were subjected to identification to provide a level of certainty that chromatographic peaks represented metabolites and to provide a picture of the nature of the effects induced by the Se congeners. For the identification of metabolites, The Human Metabolome Database (Wishart et al., 2013), also covering rat metabolites, was searched using the accurate masses of the metabolites. The presence of adducts or fragments (e.g., plus Na^+^ or minus H_2_O) at identical HPLC retention times was taken into account.

## Results

Rats dosed with Se nanoparticles or sodium selenite at 0.05 and 0.5 mg/kg bw/day for 14 days exhibited no clinical signs of toxicity and no significant decrease in body weight as compared to control. The body weights were as follows (mean ± SEM):BSA-vehicle: 171 ± 3 g; Se nanoparticles 0.05 mg/kg bw: 158 ± 3 g; Se nanoparticles 0.5 mg/kg bw: 160 ± 8 g; H_2_O-vehicle: 165 ± 4 g; sodium selenite 0.05 mg/kg bw: 164 ± 2 g; sodium selenite 0.5 mg/kg bw: 160 ± 4 g).

**Figure 2:**
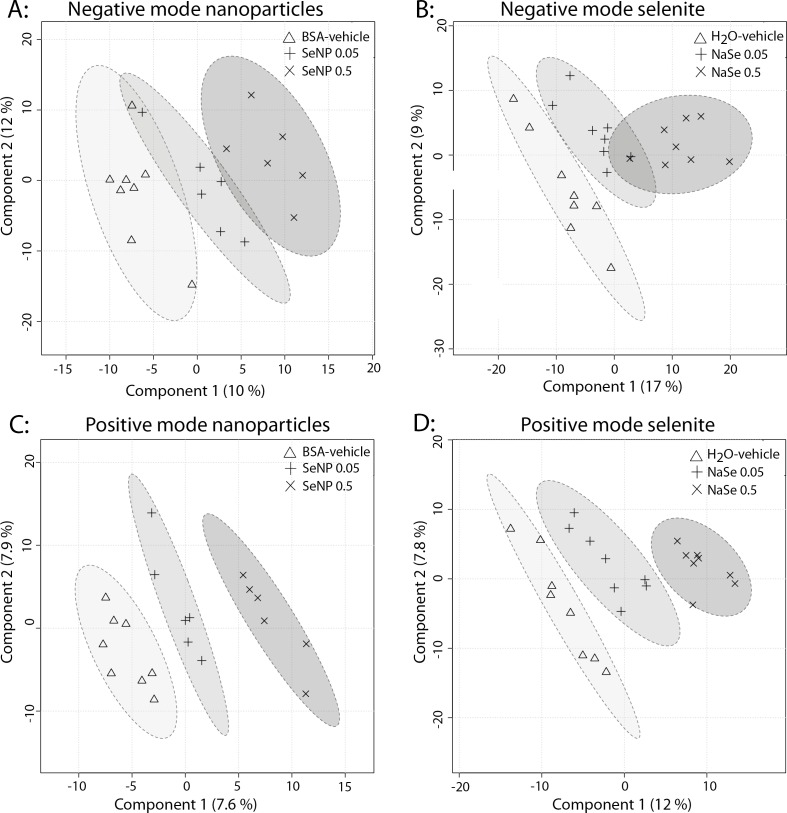
PLS-DA analyses of urine from rats administeredSe nanoparticles or sodium selenite. (A) shows Se nanoparticles in negative ionization mode, (B) selenite in negative ionization mode, (C) Se nanoparticles in positive ionization mode and (D) shows selenite in positive ionization mode. SeNP designates Se nanoparticles, and NaSe designates sodium selenite. The components designate principal components 1 and 2 of the PLS-DA analyses.

The two-dimensional graphical presentation of the results of the PLS-DA analyses on the metabolite patterns from rat urine provides a picture of the overall differences among the treatment groups (Fig. 2). In the positive mode, there were clear differences in the spatial locations of all groups, reflecting dose–response effects as indicated by the position of the 0.05 mg/kg bw/day Se group between the vehicle and the 0.5 mg/kg bw/day Se groups (Figs. 2C and 2D). In the negative mode, the picture was similar to that seen for the positive mode, although less clear (Figs. 2A and 2B).

**Figure 3:**
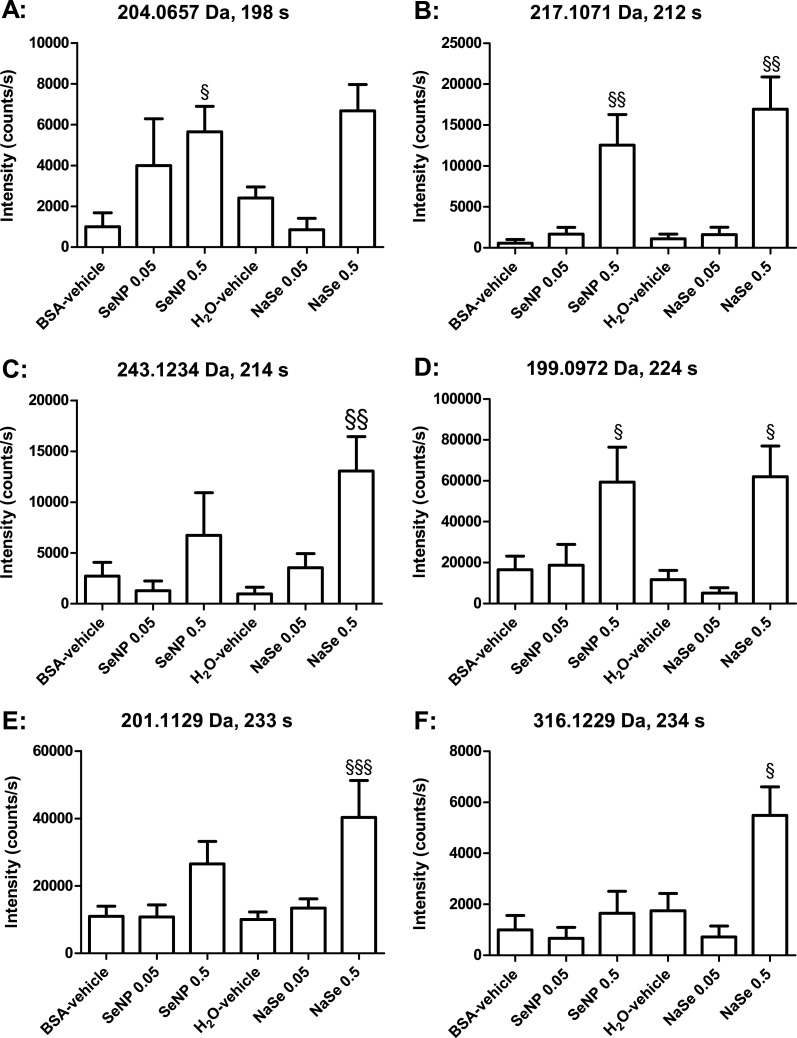
Bar graphs of individual metabolites determinedby LC-MS in negative ionization mode. SeNP designates Se nanoparticles, and NaSe designates sodium selenite. The data are the mean values, and the bars indicate SEM. The data were statistically tested as BSA-vehicle *vs.* Se nanoparticles at 0.05 and 0.5 mg Se/kg bw/day and as H_2_O-vehicle *vs.* sodium selenite at 0.05 and 0.5 mg Se/kg bw/day. The statistical tests were Kruskal–Wallis with Dunn’s post-test for data that were not normally distributed (^§^ designates *p* < 0.05, ^§§^ *p* < 0.01 and ^§§§^ *p* < 0.001).

**Figure 4:**
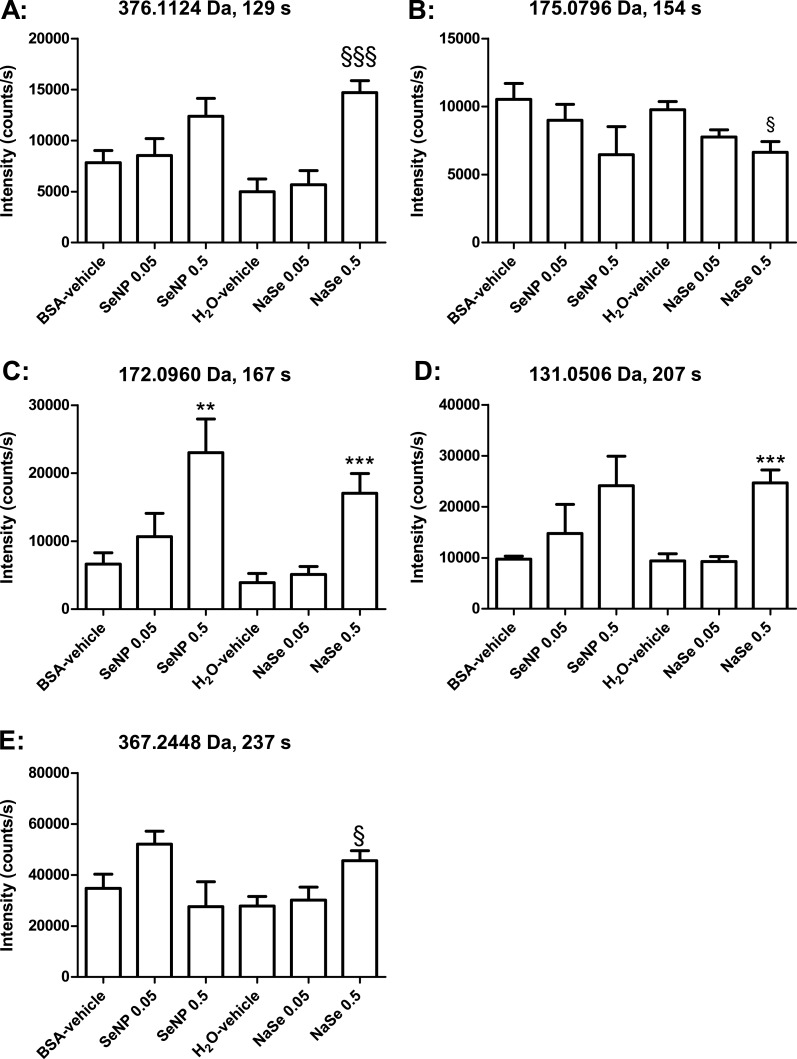
Bar graphs of individual metabolites determinedby LC-MS in positive ionization mode and indicated by their accurate masses. SeNP designates Se nanoparticles, and NaSe designates sodium selenite. The data are the mean values, and the bars indicate SEM. The data were statistically tested as BSA-vehicle *vs.* Se nanoparticles at 0.05 and 0.5 mg Se/kg bw/day and as H_2_O-vehicle *vs.* sodium selenite at 0.05 and 0.5 mg Se/kg bw/day. The tests were one-way ANOVA with Dunnett’s post-test for normally distributed data (* designates *p* < 0.05, **p < 0.01 and ***p < 0.001), or Kruskal–Wallis with Dunn’s post-test for data that were not normally distributed (^§^ designates *p* < 0.05, ^§§^ *p* < 0.01 and ^§§§^ *p* < 0.001).

Concomitant analyses of all groups using the MetaboAnalyst online tool indicated statistically significant changes in eight peaks in the negative mode and nine in the positive mode (Tables 1 and 2). Some of these were fragments of others (see Tables 1 and 2), and after accounting for these, six metabolites were significantly changed in the negative mode and seven in the positive mode. Two metabolites were detected in both negative and positive mode, and were thus only depicted in the graphs for negative mode (Figs. 3 and 4). Several metabolites showed dose–response relationships as suggested by the graphs. However, application of the post-test identified statistically significant effects only for the 0.5 mg Se/kg bw/day groups. Overall, the Se nanoparticles and sodium selenite seemed to induce similar patterns of regulation. The levels of the effects (intensity counts) likewise seemed similar and thus corresponded to similar quantitative effects on the enhanced metabolites. The number of metabolites changed significantly in the urine from the sodium selenite-administered animals was larger than that from the Se nanoparticle-administered animals. The exact mass of discriminatory metabolites (± 5 mDa) was used as a search parameter in The Human Metabolome Database (Wishart et al., 2013). The putative identities of the metabolites are presented in Tables 1 and 2.

**Table 1:** Possible identities of metabolites measured in negative ionization mode. Potential metabolites identified by comparing m/z ratios of metabolites with m/z ratios obtained from the human metabolome (HMDB) database and also taking expected natural occurrence into consideration.

Metabolite m/z (Da)	HMDB m/z (Da)	m/z error (Da)	Ret. Time (s)	Possible identity
204.0657	204.0666	0.0009	198	Cinnamoylglycine/Indolelactic acid/3-Indolehydracrylic acid/5-Methoxyindoleacetate *[M–H]*
160.0757	n/a	n/a	198	Fragment of 204.0657 *[M–CO_2_]*
217.1071	217.1082	0.0011	212	2-Hydroxydecanedioic acid/3-Hydroxydecanedioic acid *[M–H]*
243.1234	243.1167	0.0067	214	Isoleucyl-hydroxyproline/hydroxyprolyl-leucine *[M–H]*
199.0972	199.0976	0.0004	224	^a^cis-4-Decenedioic acid/cis-5-Decenedioic acid *[M–H]*
181.0873	n/a	n/a	224	Fragment of 199.0972 *[M–H_2_O–H]*
201.1129	201.1132	0.0003	233	^a^Sebacic acid/Heptylmalonic acid/3-Methylazelaic acid *[M–H]*
316.1229	316.1303	0.0074	234	Tryptophyl-Hydroxyproline/Hydroxyprolyl-Tryptophan *[M–H]*

**Notes.**

Ret. Time, Retention time.

a Also detected in positive mode.

**Table 2:** Possible identities of metabolites measured in positive ionization mode. Potential metabolites identified by comparing m/z ratios of metabolites with m/z ratios obtained from the human metabolome (HMDB) database and also taking expected natural occurrence into consideration.

Metabolite (m/z)	HMDB MW (m/z)	Error (m/z)	Ret. Time (s)	Possible identity
376.1124	n/a	n/a	129	?
175.0796	175.0713	0.0083	154	N-Acetylasparagine/Formiminoglutamic acid *[M+H]^+^*
172.0960	n/a	n/a	167	?
131.0506	n/a	n/a	207	?
201.1098	201.1121	0.0023	222	^a^cis-4-decenedioic acid/cis-5-decenedioic acid *[M+H]^+^*
165.0912	n/a	n/a	222.	Fragment of 201.1098
137.0958	n/a	n/a	222	Fragment of 201.1098
203.1268	203.1277	0.0009	234	^a^Sebacic acid/Heptylmalonic acid/3-Methylazelaic acid *[M+H]^+^*
367.2448	n/a	n/a	237	?

**Notes.**

Ret. Time, Retention time.

? designates several possible candidates whose biological relevance is difficult to judge.

a also detected in negative mode.

## Discussion

In the present study, we investigated biological and toxicological effects of Se nanoparticles at low dose and for comparison included Se in the form of selenite. The PLS-DA analyses on rat urine indicated effects of both formulations at the low (0.05 mg/kg bw/day) and the higher (0.5 mg/kg bw/day) Se doses (Fig. 2). Among the 11 metabolites found to be significantly changed, 10 displayed similar patterns of metabolite regulation for both Se formulations. Assuming that urine reflects the excreted end products of a high proportion of the body’s metabolic processes, there is a strong indication of similar biological effects of the two different formulations. It was, however, difficult to determine the exact identity of the metabolites, although dipeptides, decenedioic acid and hydroxydecanedioic acid were likely candidates that could reflect altered energy metabolism. Both cis-4-Decenedioic acid and cis-5-Decenedioic acid have been reported as urinary products of oleic and linoleic acid oxidation (Jin & Tserng, 1990). Oleic and Linoleic are abundant fatty acid in rat adipose tissue with percentages of 27 and 42%, respectively (Ahn et al., 2010). Thus, fatty acid metabolism induction by both Se formulations could be suggested. 3-Hydroxydecanedioic acid has been identified as a major compound in urine from patients with ketoacidosis and suggested by the authors to be formed from fatty acids by a combination of omega-oxidation and incomplete beta-oxidation (Greter et al., 1980). This also suggests a disturbed fatty acid metabolism. The presence in the urine of dipeptides such as isoleucyl-hydroxyproline/hydroxyprolyl-leucine, tryptophyl-hydroxyproline/hydroxyprolyl-tryptophan could reflect disturbed protein metabolism. Notably these two dipeptides were not significantly changed for Se nanoparticles, suggesting that on this parameter the nanoparticles were actually less biologically active as compared to selenite. We previously found equal Se in blood and organs following the dosage of Se nanoparticles and selenite to rats for 28 days in the animals of the current study. This together with the finding that the high doses of both forms of Se were equally available for incorporation into selenoprotein P suggests that the bioavailability of Se from both formulations was in a similar range (Loeschner et al., 2014). Data from others are also in line with this. Similar effects of Se nanoparticles and sodium selenite on iron, transferrin and on neutrophils have been observed in sheep (Kojouri et al., 2012a; Kojouri et al., 2012b). In contrast, a higher biological effect of Se as selenite ions as compared to nanoparticles has been reported in other studies. For some toxicity endpoints, selenite was more potent than Se nanoparticles in mice and rats (Zhang et al., 2001; Zhang et al., 2005; Jia, Li & Chen, 2005; Benko et al., 2012).

In conclusion, we used a metabolite pattern approach to investigate the biological effect profiles of Se nanoparticles compared to sodium selenite at low doses. Both formulations had similar effects on a range of metabolites. Identified likely metabolite candidates included increased decenedioic acid and hydroxydecanedioic acid for both Se formulations whereas dipeptides were only increased for selenite. These effects could reflect altered fatty acid and protein metabolism, respectively.

## Supplemental Information

10.7717/peerj.2601/supp-1 Data S1 Raw metabolomics data - negative mode

10.7717/peerj.2601/supp-2 Data S2 Raw metabolomics data - positive mode

## Additional Information and Declarations

### Data Availability

The following information was supplied regarding data availability: The raw data in the form of chromatogram peak intensities have been supplied as Data S1 and S2.
